# Design of Artificial Riboswitches as Biosensors

**DOI:** 10.3390/s17091990

**Published:** 2017-08-30

**Authors:** Sven Findeiß, Maja Etzel, Sebastian Will, Mario Mörl, Peter F. Stadler

**Affiliations:** 1 Bioinformatics Group, Department of Computer Science, and Interdisciplinary Center for Bioinformatics, University Leipzig, Härtelstraße 16–18, 04107 Leipzig, Germany; sven@bioinf.uni-leipzig.de (S.F.); will@tbi.univie.ac.at (S.W.); studla@bioinf.uni-leipzig.de (P.F.S.); 2 Faculty of Computer Science, Research Group Bioinformatics and Computational Biology, University of Vienna, Währingerstraße 29, A-1090 Vienna, Austria; 3 Faculty of Chemistry, Department of Theoretical Chemistry, University of Vienna, Währingerstraße 17, A-1090 Vienna, Austria; 4 Institute for Biochemistry, Leipzig University, Brüderstraße 34, 04103 Leipzig, Germany; maja.etzel@uni-leipzig.de; 5 German Centre for Integrative Biodiversity Research (iDiv) Halle-Jena-Leipzig, 04103 Leipzig, Germany; 6 Max Planck Institute for Mathematics in the Sciences, Inselstraße 22, 04103 Leipzig, Germany; 7 Fraunhofer Institute for Cell Therapy and Immunology, Perlickstrasse 1, 04103 Leipzig, Germany; 8 Center for RNA in Technology and Health, University of Copenhagen, Grønnegårdsvej 3, 1870 Frederiksberg, Denmark; 9 Santa Fe Institute, 1399 Hyde Park Road, Santa Fe, NM 87501, USA

**Keywords:** aptamer, RNA structure, ligand binding, refolding, thermodynamics, rational design, folding kinetics

## Abstract

RNA aptamers readily recognize small organic molecules, polypeptides, as well as other nucleic acids in a highly specific manner. Many such aptamers have evolved as parts of regulatory systems in nature. Experimental selection techniques such as SELEX have been very successful in finding artificial aptamers for a wide variety of natural and synthetic ligands. Changes in structure and/or stability of aptamers upon ligand binding can propagate through larger RNA constructs and cause specific structural changes at distal positions. In turn, these may affect transcription, translation, splicing, or binding events. The RNA secondary structure model realistically describes both thermodynamic and kinetic aspects of RNA structure formation and refolding at a single, consistent level of modelling. Thus, this framework allows studying the function of natural riboswitches in silico. Moreover, it enables rationally designing artificial switches, combining essentially arbitrary sensors with a broad choice of read-out systems. Eventually, this approach sets the stage for constructing versatile biosensors.

## 1. Introduction

**Figure 1:**
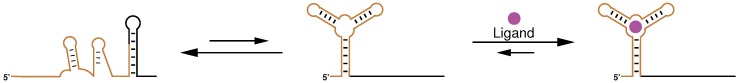
Illustration of the general riboswitch mechanism. Typically, two structural alternatives (**left** and **middle**) dominate the ensemble of a riboswitch sequence. The one that contains the correctly folded aptamer structure (**middle**) is further stabilized upon ligand addition and the system gets trapped in the bound conformation (**right**).

Non-protein-coding RNAs regulate diverse cellular processes, including the fundamental steps of protein production transcription and translation [1]. Commonly, this function is closely connected to the structure. An important class of such RNA elements are riboswitches, which typically reside within the 5${}^{\prime }$-untranslated region (UTR) of prokaryotic protein coding transcripts. Located within this non-coding regulatory region, riboswitches are able to directly influence the process of transcription and translation by forming structural alternatives, Figure 1. The first can be controlled by the formation of transcription terminators, which are—in essence—stable hairpin loops followed by a series of uracil nucleotides (nts). The efficiency of translation initiation in bacteria is highly dependent on the accessibility of two sequence motifs: (i) the ribosome bidning site (RBS), responsible to recruit the ribosome complex, and the start codon, mainly AUG, of the downstream protein coding region; both can be sequestered by local RNA structures. On this basis, riboswitches easily link manifold environmental changes to either the formation of terminator structures or the accessibility of sequences important for efficient translation. Natural riboswitches sense the concentration of small ligands such as enzyme cofactors (SAM and TPP), nucleotide precursors (guanine, adenine and 2${}^{\prime }$-deoxyguanosine), amino acids (lysine) and metal ions (magnesium) [2]. Moreover, changes in temperature [3] and pH-value [4] are sensed by riboswitches. As these regulatory elements are encoded on messenger RNA (mRNA) level they give access to directly alter protein expression at an early stage, which saves resources and is potentially faster than regulation by proteins such as transcription factors, which need to be produced on demand.

Going beyond naturally evolved riboswitches, it is highly attractive to design analogous, but artificial, regulatory RNA elements, which respond to arbitrary environmental changes, e.g., sensing small molecules. Aptamers against any small molecule of interest can—at least in principle—be selected utilizing the SELEX (Systematic Evolution of Ligands by Exponential enrichment) protocol [5,6]. A rational riboswitch design process on this basis paves the way for manifold applications. For instance in white biotechnology, where heterologous or synthetic pathways are implemented in a host organism to produce specific chemicals from renewable resources, it is essential to sense and react on the accumulation of presumably toxic intermediates [7]. Furthermore, it has been shown that riboswitches can function as biosensors with medical applications and to modulate cell behavior such as motility (recently reviewed in [8]). In this context, synthetic constructs are of particular interest [9,10]. Synthetic riboswitches based on artificial aptamers are particularly valuable gadgets because they can respond to ligands that are orthogonal to the host organism’s native metabolites and thus can be triggered without affecting vital functions of the host. Theophylline riboswitches may serve as an example [11]. The corresponding aptamer as well as other—artificial or naturally occurring—ligand-binding RNA structures are used as components in a whole variety of different conceptual principles in the design of synthetic riboswitches. A detailed description of these RNA regulators is given in several recent reviews [12,13,14,15]. In addition, there no guarantee that there exists a naturally evolved riboswitches that is sensitive to a ligand of interest in an engineering context, such as the design of a drug sensor.

In this contribution we first review essential components of riboswitches and read-out mechanisms from experimental perspective. We then focus on thermodynamic and kinetic aspects to de novo design or further investigate riboswitches in silico. Finally, recent design attempts are briefly outlined and graphically summarized.

## 2. Components

### 2.1. RNA Aptamers

Aptamers are RNA (or less relevant for us in the present context, DNA) molecules that specifically bind to target molecules. Over the last 25 years, aptamers for a wide variety of targets have been generated by SELEX. Similar to natural aptamers found in riboswitch elements, those artificial aptamers often have sensitive and specific ligand recognition properties [16,17].

Suitable aptamers for sensor design must react to ligand binding with sufficient change in structure and/or stability. The basic principle is that the response of the aptamer to ligand binding propagates along the RNA construct and eventually causes structural changes at sites possibly far away from the ligand binding pocket. It can be this distal structural effect that triggers the desired readout. A key problem in the design of synthetic riboswitches is that the necessary prerequisites on the aptamer are by no means fully understood. Therefore, only a few in vitro selected RNA aptamers were successfully integrated in functional riboswitch constructs so far.

A frequently used SELEX-derived aptamer in synthetic riboswitches is the theophylline-binding aptamer TCT8-4, isolated by Jenison et al. [18]. This aptamer has a high discriminatory potential against structurally closely related purines like caffeine, which differs from theophylline only by a single methyl group at position N${}_{7}$. Yet, the discrimination is 10,000-fold. With an aptamer binding constant of 320 mM [18,19] and good cell permeability, theophylline represents an excellent ligand for synthetic riboswitches, despite its cytotoxicity at higher concentrations [11,18,20,21,22,23]. While the ligand-free structure of the aptamer is rather dynamic and adopts several different conformations, binding of theophylline induces a structural rearrangement, stabilizing a highly defined and robust structure [19,24,25,26].

Another aptamer identified by SELEX successfully used in riboswitch-mediated regulation of translation is the tetracycline-binding aptamer [27,28,29,30]. Similar to the theophylline aptamer, the tetracycline-unbound state of the RNA exists in a less stable pre-formed scaffold, and ligand binding induces a conformational rearrangement resulting in a more compact structure [26,31,32,33,34]. As a result, the interaction of the RNA with tetracycline shifts the thermodynamic equilibrium from the ligand-free structure towards the tetracycline-bound structure [26,32].

A conformational change of the ligand-bound aptamer structure may be a prerequisite for its riboswitch compatibility in vivo [26,35,36]. This hypothesis is further supported by the investigation of riboswitches based on neomycin-binding aptamers [37,38]. Here, a SELEX-derived aptamer R23 shows a high affinity for its ligand. Yet, when cloned in front of the reporter gene, no ligand-dependent regulation is observed. This can be explained by the fact that R23 folds into a stable pre-formed ligand-binding pocket even in the absence of neomycin. A second neomycin aptamer, N1, was selected in an in vivo screen. Without ligand, this aptamer forms an unstable structure, and only the binding of neomycin induces the rearrangement of the RNA into a stable conformation, blocking the scanning ribosomes on the mRNA, and, consequently, translation [37,39].

An alternative strategy to generate orthogonal riboswitches is to re-engineer natural aptamer domains. Using randomization by site-directed mutagenesis with genetic selection, the Micklefield lab reprogrammed natural riboswitches to bind non-natural synthetic ligands, while the original intracellular target molecules were no longer recognized. The parental ON and OFF riboswitches were an adenine-sensing translational ON switch from *Vibrio vulnificus* and a transcriptional OFF switch from *Bacillus subtilis*, recognizing the queuosine precursor preQ${}_{1}$ [40,41]. Both were re-engineered into orthogonal selective riboswitches specific for non-natural purine derivatives, while the regulatory principle of these switches remained unchanged.

### 2.2. Read-Out Mechanisms

To monitor the functionality of artificial riboswitches, suitable read-out systems are required. A plethora of different reporter systems are available and are frequently used in the qualitative and quantitative analysis of riboswitch-mediated gene regulation. Consequently, this text can provide only a short overview of the main read-out systems.

Most frequently, reporter systems based on protein level are used, like the green fluorescent protein (GFP) or variants thereof in both pro- and eukaryotes [22,37,42]. Another very common reporter protein is the bacterial *lacZ* gene, encoding for β-galactosidase [43,44]. The enzymes activity can be used for a color-based screen of the resulting individual bacterial colonies, allowing at least a qualitative evaluation. For a quantitative analysis, the relative fluorescence of GFP can be directly determined and for β-galactosidase, the enzymatic activity in the cell extract can be measured by the turn-over of nitrophenyl-β-d-galactopyranoside (ONPG), resulting in the cleavage product *o*-nitrophenol that can be quantified by its absorption at 420 nm.

Furthermore, genes conferring resistance against antibiotics allow a growth-dependent analysis of riboswitch-mediated regulation. The possibility to use the tetracycline-resistance mediating *tetA* gene for positive as well as negative selection modes renders this reporter a very versatile selection system. When *tetA* is under riboswitch control, cells survive in the presence of tetracycline only if the gene is induced by the regulator. To counter-select for riboswitch-independent gene expression, the surviving cells are cultivated in nickel-containing medium, as the *tetA*-encoded efflux pump leads to an increased cell permeability to toxic Ni${}^{2+}$ ions [45,46,47,48].

A very different, yet effective reporter system for riboswitch activity was introduced in [49]. The chemotactic movement of *E. coli* is controlled by an intracellular phosphorylation/dephosphorylation cascade that transfers the signal of an attractant bound to a cell surface receptor to the flagellar motor [50]. In the phosphorylated state, the last component of the cascade, CheY, binds to the motor proteins and forces the flagellum to perform clockwise rotations, resulting in cell tumbling. CheZ, a phosphatase, dephosphorylates CheY. As a consequence, CheY leaves the flagellar motor, and the flagellum rotates counter-clockwise, allowing the bacterium to swim smoothly. When under the control of a theophylline-sensing riboswitch, CheZ is only synthesized in the presence of this ligand, and the induced cells exhibit a movement towards a theophylline source. This induced cell motility allowed the separation of cells harboring a functional theophylline-dependent riboswitch (motile) from those that carried a non-functional construct (immotile) [49,51].

As the described read-out systems are all based on the expression of selectable proteins, they are predominantly suited to monitor riboswitches controlling translation. Nevertheless, they can also be used for the analysis of transcriptional riboswitches, although they do not allow for a direct read-out of transcription efficiency. Unexpected side effects have to be considered that can have an impact on translation, leading to an increased read-out signal, without any increase in transcription [23,52]. Hence, for riboswitches acting on transcription, direct RNA sensing systems are better suited.

A simple direct and frequently used detection method of mRNA expression is Northern blot analysis. Due to a rather elaborate and time-consuming detection, this analysis is only rarely applied in riboswitch research [23]. A more convenient approach is a direct optical read-out of RNA molecules. Instead of GFP and its variants, fluorescent RNA aptamers can be used as reporters. The SELEX-derived Spinach aptamer is an RNA mimic of GFP, forming a similar fluorophore in its binding pocket when interacting with its ligand 3,5-difluoro-4-hydroxybenzylidene imidazolinone (DFHBI) [53]. Similar to GFP, Spinach variants with different colors could be generated. The Spinach aptamer was successfully used to monitor metabolite binding to natural riboswitch aptamers [54,55,56,57,58]. A second fluorescent DFHBI-binding aptamer is Broccoli, also isolated by SELEX, but optimized for in vivo applications by cellular screening [59]. In 2014, the Unrau lab developed a third fluorescent aptamer that is structurally unrelated to Spinach or Broccoli [60]. The Mango aptamer binds thiazole orange (TO1) with high affinity and was used to monitor expression of 6S RNA in *E. coli*, where it was inserted into the corresponding gene. Due to its higher fluorescent efficiency compared to Spinach [60], the Mango aptamer seems to represent a very promising alternative approach for direct quantification of riboswitch-controlled RNA synthesis. Finally, the Jäschke lab designed combined fluorophore and quencher molecules, where either the fluorophore or the quencher is recognized by an in vivo expressed aptamer. As a result, fluorophore and quencher are separated and lead to a significant increase in fluorescence [61,62]. Such quencher- or fluorophore-binding RNA domains might represent useful read-out systems for monitoring the activity of transcription-regulating riboswitches. However, compared to the read-out based on fluorescent protein expression, the fluorescent RNA domains, have a considerable disadvantage. In translation, many copies of GFP are synthesized per mRNA, increasing the fluorescent signal dramatically. In contrast, a fluorescent RNA domain is synthesized only once per RNA transcript, and the resulting signal is very low. Yet, the sensitivity can be increased by introducing serial arrangements of fluorescent aptamer regions, as it was shown for Broccoli [59] or for Spinach [63]. In case of Spinach, tandem arrays of 8–64 aptamer repeats were tested.

The choice of translation- or transcription-based readout affects the computational design strategy. The control of translation is reversible since the secondary structure at the translation initiation site can switch from accessible to inaccessible conformations many times during the life-time of an mRNA. Translational switches are thus primarily controlled by the equilibrium thermodynamics of RNA structure formation. Control at the transcriptional level, in contrast, determines whether a complete or an incomplete mRNA is produced. In the OFF state of the switch this requires the formation of a pre-mature termination signal to be formed in the nascent transcript. In the ON state, no premature termination is induced and a complete mRNA is transcribed. This mechanism depends crucially on the interplay of transcription and structure formation and thus is controlled to large extent by co-transcriptional kinetic effects of RNA folding.

An interesting variation on the theme is to link ligand binding to (alternative) splicing rather than the control of translation. This has been observed for instance for the binding of the protein PKR to specific structures in the UTRs of some cytokine mRNAs; this leads to structural changes in the mRNAs that in turn affect splicing [64]. As in the transcription-based riboswitches the control is irreversible and may be controlled at least in part kinetically.

### 2.3. Composition of Functional Switches

A typical riboswitch consists of a sensory domain (natural or synthetic aptamer) that is linked to a regulatory domain (expression platform). Ligand binding to the aptamer induces a conformational change in the expression platform, which affects translation or transcription of the downstream located gene. In the case of translation regulation, the expression platform contains regulatory motifs, e.g., a RBS or AUG, that—upon ligand binding—are sequestered in base-pairing (OFF switch) or presented and accessible for the ribosome (ON switch). In transcriptional riboswitches, the expression platform consists of a terminator/antiterminator structure that controls RNA polymerization. Similar to the translational riboswitches, ligand binding in the aptamer domain induces a structural rearrangement. This leads to the formation of the terminator structure that shuts off transcription (OFF switch) or an alternative structure (antiterminator) that allows the RNA polymerase to proceed with transcription, resulting in gene expression (ON switch). In such cases, a clear distinction between sensory domain and a regulatory domain is often not possible, see for instance the exemplified designs in Section 4.1 and Section 4.3.

While the ribosomal binding site represents a nucleotide sequence that is directly recognized by the small ribosomal subunit, the terminator consists of a combination of secondary structure and sequence. The consensus form of a typical intrinsic terminator has a GC-rich palindromic sequence that—when transcribed—folds into a stable hairpin with 5 to 15 base pairs, carrying a stable tetraloop. This structure is followed by a polyU tract of approximately 8 to 11 residues [65,66,67]. After transcribing the terminator, the RNA polymerase pauses at the U stretch region. Subsequent formation of the hairpin in the nascent RNA within the polymerase weakens the hybrid interaction between DNA template and synthesized RNA, leading to inactivation of the transcription complex and destabilization of the RNA/DNA hybrid [65]. In the U stretch region, the rU/dA hybrid strongly contributes to the dissociation of the transcription complex, as these base pairs between DNA and RNA are the least stable ones [68]. The consensus composition of a terminator does not necessarily correlate with its termination efficiency [23]. Additional features, like flanking sequences, may play a role as well, especially if aptamer elements are present in a riboswitch context, where competing secondary structures and folding traps are possible [69]. As a consequence, functionality and efficiency of terminators are hard to predict, and a stable hairpin element is not always a good terminator. In a transcriptional riboswitch, it is more important that the hairpin stability is similar to that of the aptamer fold, in order to allow a ligand-dependent refolding of the riboswitch. It is possible that for each riboswitch construct, the terminator part has to be adjusted to the aptamer not only in terms of sequence composition, but also in its stability [23,69].

Taken together, the implementation of a ribosomal binding site as a riboswitch expression platform is rather straight forward, as predominantly masking interactions of this short sequence with the aptamer and the refolding of the mRNA transcript have to be considered. In contrast, the design of transcriptional riboswitches is a more complex task, where—in addition to a defined primary sequence (U stretch)—secondary structure elements (hairpin structure) and the local arrangement of both are critical. It is important to note, however, that the rho-independent termination is not necessarily ubiquitously present in bacteria, and even if this should turn out to be the case, there seem to be substantial variations in the structure and organization of termination signals [70]. Consequently, there is no guarantee that an artificial, transcription regulating riboswitch that works efficiently in *E. coli* also functions properly in distant species such as *Helicobacter* or *Synechocystis*, or *Mycoplasma*.

Besides the described cis-regulatory riboswitch elements, there exists a whole variety of systems, where regulatory RNAs act in trans; for example, RNA switches that activate or repress transcription by interaction with an antisense RNA [71]. The design of regulatory RNAs controlling gene expression was described in *E. coli* [72], where the theophylline-binding aptamer was coupled to a small RNA (sRNA). Regulation occurs via allosteric interaction, masking or releasing the sRNA part upon ligand binding. If the sRNA is not involved in base-pairing with the aptamer domain, it functions as a trans-acting regulatory RNA. In an alternative approach, regulation of gene expression is mediated by ligand-dependent ribozyme cleavage of a target mRNA. In such aptazyme-based riboswitches, the hammerhead ribozyme (HHR) is a frequently used expression platform. Ogawa and Maeda fused this ribozyme to the theophylline aptamer [73,74]. As a result, the self-cleavage activity of the ribozyme was controlled by the interaction of the aptamer domain with its ligand. Integrating this construct into a regulatory platform, consisting of a RBS and a complementary anti-RBS, resulted in an artificial riboswitch: the ligand-dependent HHR-mediated cleavage reaction removed the anti-RBS domain from the transcript, releasing the RBS and thus enabling translation of the mRNA in vitro as well as in vivo. A second ribozyme that was integrated into a regulatory RNA element is the hepatitis delta virus ribozyme. Insertion of an aptamer domain (the SELEX-derived theophylline aptamer or the guanine-binding domain of a naturally occurring riboswitch from *B. subtilis*) into this ribozyme resulted in an RNA structure that—when inserted into the 3${}^{\prime }$-UTR of a eukaryotic GFP mRNA—regulated mRNA stability and, as a consequence, gene expression in a ligand-dependent way [75]. The theophylline- and the guanine-sensing constructs could be combined in a tandem arrangement that functions as a logic NOR gate.

Recently, also one of the newly discovered catalytic RNA molecules, the twister ribozyme [76], was successfully inserted into a synthetic riboswitch [77]. The Hartig group fused this ribozyme to aptamers recognizing theophylline or TPP and inserted these constructs into the 5${}^{\prime }$-UTR of a GFP mRNA in *E. coli*, where they could observe a ligand-induced cleavage of the mRNA, regulating the accessibility of the ribosomal binding site in both ON- as well as OFF switch designs. Similar to the above mentioned HHR-based aptazyme, they generated a regulatory RNA cleavage module containing a neomycin aptamer in the 3${}^{\prime }$-UTR of a yeast, where ribozyme-mediated cleavage removed the poly A tail of the mRNA, reducing its stability. An elegant combination of several twister-based riboswitch devices resulted in a series of Boolean logic AND, NAND, OR, NOR and ANDNOT gates [77].

## 3. Theory

### 3.1. Thermodynamics of RNA Folding

RNA folding is dominated—in good approximation—by the formation of secondary structures, i.e., Watson-Crick and GU base pairs. While tertiary interactions of course play a key role for the actual function, they have less impact on thermodynamics of the folding process. A wealth of empirical data, amassed over the last decades, has shown that the energy of an RNA secondary structure is very well approximated by additive, sequence-dependent contributions of stacked base pairs and looped regions. This is known as the Turner energy model [78].

RNA secondary structures are simple combinatorial objects: they are defined as sets of base pairs satisfying three simple conditions: (i) each base can contribute to at most one base pair, which must be a GC, AU, or GU pair; (ii) base pairs do not cross, i.e., the structure does not contain any pseudoknot; and (iii) hairpin loops comprise at least three unpaired bases. Consequently, secondary structure prediction is phrased as a combinatorial optimization problem: among all secondary structures, find the one that minimizes the energy as determined by the Turner model.

This optimization problem can be solved—optimally and efficiently—by dynamic programming in $O(n^{2})$ space and $O(n^{3})$ time, where *n* is the length of the RNA sequence. Several widely-used software packages are available for RNA folding, most prominently mfold/UNAfold [79,80] and the Vienna RNA package [81,82]. We refer to [83,84,85] for a discussion of the accuracy of RNA secondary structure prediction and alternative energy models.

The recursive approach allows not only to solve the energy minimization problem, but also to compute partition functions [86], thus providing access to all relevant thermodynamic parameters of the entire ensemble of possible secondary structures, including specific heat and melting temperatures.

The secondary structure model is applicable to predicting the structures of both individual and interacting RNA molecules. In this context the “no-crossing rule” adhered to by the simplest RNA–RNA interaction models [87] (implemented e.g., in RNAcofold [88], NUPACK [89]) become more restrictive and excludes also common motifs such as kissing hairpins. More elaborate algorithms, however, can overcome this limitation to a certain extent, see e.g., [90,91,92,93]. For the sake of exposition, we restrict ourselves to interactions of two RNA partners. In simple cases of interacting structures, e.g., the one covered by RNAcofold, the required partition functions can be computed efficiently.

In contrast to the folding of single RNAs, RNA–RNA interactions are bimolecular; thus, their thermodynamics and kinetics explicitly depend on concentrations. The equilibrium constant for the formation of RNA duplexes can be computed directly from the partition functions:

(1) $$K=\frac{\left[AB\right]}{\left[A]\;[B\right]}=\frac{Z_{AB}}{Z_{A}\;Z_{B}},$$

where $Z_{A}$ and $Z_{B}$ are the partition functions for the ensemble of secondary structure of unbound *A* and *B* RNAs, and $Z_{AB}$ is the partition function over all bound AB complexes. The latter can be computed either directly [89] or it can be obtained from the partition function $Z_{AB}^{\prime }$ of all bound and unbound states by subtracting the partition function $Z_{A}Z_{B}$ of the non-interacting pairs. Since the bound states are subject to an extra initialization energy ϵ [94] we have

(2) $$Z_{AB}=e^{-\epsilon /RT}\left(Z_{AB}^{\prime }-Z_{A}Z_{B}\right),$$

where *R* and *T* are the Boltzmann’s constant and temperature in Kelvin, respectively. Together with the usual mass balance conditions $\left[A\right]+\left[AB\right]=a_{0}$ and $\left[B\right]+\left[AB\right]=b_{0}$ in Equation (1), the RNA secondary structure model provides complete information on interacting RNAs. This basic idea immediately extends to networks of RNA–RNA interactions, since binding constants for interactions of other RNA species, including larger complexes, can be computed analogously [89].

**Figure 2:**
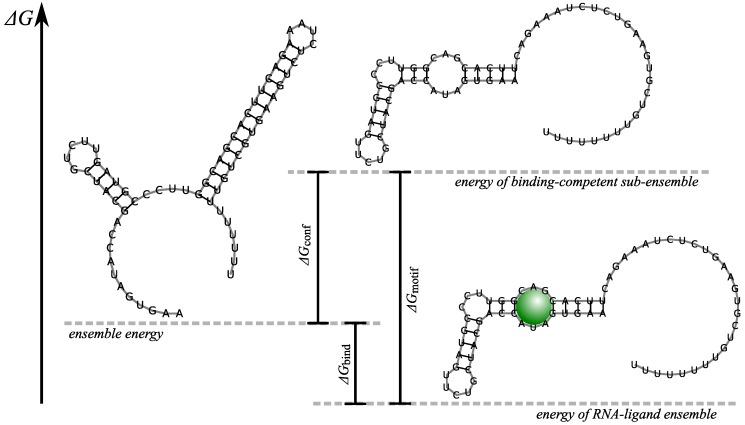
Ligand binding and structural stabilization of the aptamer—schematic illustration based on the theophylline aptamer. The sub-ensemble of binding competent structures (**middle**) is raised over the unbound structure ensemble (**left**) by the energy $\Delta G_{\mathrm{conf}}$ required for the formation of the binding pocket. Due to the RNA–ligand binding energy $\Delta G_{\mathrm{motif}}$ the RNA–ligand complex (**right**) is stabilized over the unbound structures by the energy $\Delta G_{\mathrm{bind}}$. Note that the ensembles are simply visualized by representative structures—while consisting of many secondary structures.

The same thermodynamic approach can be used to model RNA–ligand interactions with non-RNA ligands like small RNA-binding molecules or proteins. However, this requires additional empirical information. First a model of the binding site is required that is then translated into a constraint on RNA secondary structure level. In the simplest case, which covers already the majority of RNA binding proteins, one requires that the binding site has a high probability to be unpaired and shows a particular sequence pattern; a prominent example is the RBS. The probability that a certain sequence interval is unpaired can be computed e.g., with RNAup [90,95] or IntaRNA [91]. The probability that a particular sequence motif is present in a structural context, e.g., partially structured binding site in the equilibrium ensemble, can also be obtained computationally e.g., using the constraint framework implemented in the ViennaRNA package [96]. In either case one computes, in addition to the total partition function *Z*, the constrained partition function $Z^{*}$ that enforces the presence of the required structural motif. This allows calculating its probability $p^{*}=Z^{*}/Z$ and the refolding energy $\Delta G_{\mathrm{conf}}=-RT\ln p^{*}$ that is required to force the ensemble into the binding conformation. This simple relationship was tested experimentally already a decade ago for the binding of the HuR to unpaired RNA sequence motifs [97]. The total binding energy can thus be modeled as

(3) $$\Delta G_{\mathrm{bind}}=\Delta G_{\mathrm{conf}}+\Delta G_{\mathrm{motif}},$$

where $\Delta G_{\mathrm{motif}}$ is the binding energy of the ligand to an ideally structured binding site. The latter has to be determined empirically and will in general strongly depend on the sequence. For interactions with small ligands, one pragmatically assumes that the sequence of the native binding site is necessary and must form a coherent unit, modeled as specific loop, in any secondary structure that binds the ligand. In this approximation only a single empirical energy parameter $\Delta G_{\mathrm{motif}}$ needs to be determined, Figure 2.

### 3.2. Kinetic of RNA Folding

RNA secondary structure as model system has the key advantage that kinetic effects can be studied consistently with the thermodynamic considerations outlined in the previous section. To this end, one defines the energy landscape of a given RNA that consists of all possible secondary structures *x* of the RNA together with their energies E(x) in the underlying thermodynamic model; two secondary structures are adjacent if they differ by the opening or closing of a single base pair. Since each secondary structure represents an equivalence class of actual 3D conformations, the insertion or deletion of a base pair approximates the actual motion of the molecule in continuous, three-dimensional space. In other words, the dynamics of RNA folding and the transitions between different structural states are modeled as Markov processes on this energy landscape. Transition rates between adjacent states are naturally approximated as Arrhenius rates of the form

(4) $$r_{y\leftarrow x}=k_{0}e^{-(E_{xy}^{\neq }-E\left(x\right))/RT}.$$

The free energy of the transition state $E_{xy}^{\neq }=\max\left\{E\left(x\right),E\left(y\right)\right\}+\epsilon^{\neq }$ depends on the energies of the respective secondary structures *x* and *y* before and after insertion/deletion of a single base pair and the activation energy $\epsilon^{\neq }$, which models the energy barrier encountered when rotating a base pair in or out of a helix; moreover, $k_{0}$ is an entropic term that gauges the time scale. The corresponding Markov process is ergodic and satisfies detailed balance. It is easy to verify that its equilibrium distribution is exactly the Boltzmann equilibrium distribution p(x)=exp(−E(x)/RT)/Z.

This system is most straightforwardly studied by directly simulating (re)folding trajectories [98,99]. The required multitude of trajectories at elementary step resolution, makes these approaches computationally very expensive. At the same time the space of RNA secondary structure is by far too large to treat the Markov chains analytically at this resolution. For example, since the secondary structure space grows exponentially, already sequences of length 30 can form more than $10^{6}$ secondary structures, which prohibits direct analytic approaches. To deal with interesting large systems, coarse-grained representations that drastically reduce the number of representative structures have been investigated. These fall into two large classes: (i) coarse-grained representations of secondary structures with associated coarse-grained transition rules and (ii) partitioning of the energy landscape into ‘basins’ of kinetically related conformations. The best-studied instances of the first class are helix-based models [100,101,102,103]. While these approaches drastically reduce the search space, it is not clear *a priori* to what extent structures constructed from a restricted set of helices are the ones that are most important for the folding kinetics. In addition, it is not trivial to construct good approximations for the transition rates between these structures. A recent model that uses shape abstractions [104] incurs similar difficulties. There is no guarantee that shape representatives (shreps) are a good approximation to the set of local minima with the same abstract shape; moreover, —similar to the helix-based approaches—there is no canonical way to construct the transition rates. The alternative approach assigns each secondary structure *s* to a macrostate α, and makes the approximation that trajectories are equilibrating within each macrostate before leaving it again. Most commonly, macrostates are composed of states having gradient descent paths to the same local minimum in the landscape. In this way, the landscape is partitioned into the (gradient) basins of attraction of its local minima. Assuming equilibrium within each basin, transition rates between these macrostates can be derived [105]:

(5) $$r_{\beta \leftarrow \alpha }\;=\;\frac{1}{Z_{\alpha }}\sum_{\begin{array}{c} x\in \alpha \end{array}y\in \beta }r_{y\leftarrow x}e^{-E(x)/RT}\;=\;k_{0}Z_{\alpha \beta }^{\neq }/Z_{\alpha }\;=\;k_{0}e^{-(E_{\alpha \beta }^{\neq }-E\left(\alpha \right))/RT},$$

where $Z_{\alpha }$ is the partition function constrained to the macrostate α, $E\left(\alpha \right)=-RT\ln Z_{\alpha }$, and the free energy of the transition state is $E_{\alpha \beta }^{\neq }=-RT\ln Z_{\alpha \beta }^{\neq }$, where $Z_{\alpha \beta }^{\neq }=\sum_{x\in \alpha ,y\in \beta }e^{-E_{xy}^{\neq }/RT}$ is the partition function of the macro-transition state from α to β.

For gradient basins these quantities can be computed by combining the enumeration [106] or sampling [107] of suboptimal secondary structures with a flooding algorithm [98,108] at least for moderate-sized systems. Large systems can be handled by using the Arrhenius approximation for $r_{\beta \leftarrow \alpha }$ based on low energy paths between the local minima α and β. Non-zero transition rates $r_{\beta \leftarrow \alpha }$ can be restricted further in the barrier tree [109,110,111,112] or in the basin hopping graph [113], which enables analyzing larger RNAs.

**Figure 3:**
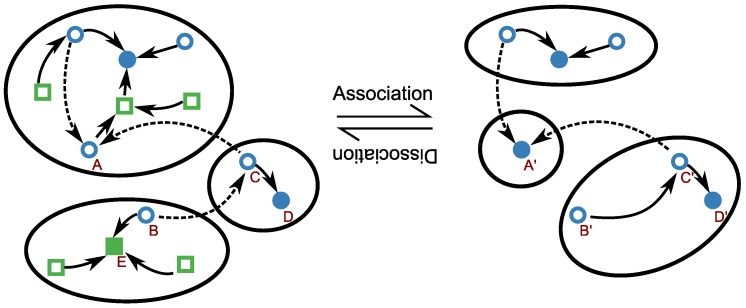
Energy landscapes of the coarse-grained RNA–ligand kinetic approach. There is a simple correspondence between the energy landscapes of monomers (**left**) and dimers (**right**): all secondary structures are states of the monomer landscape, whether they are binding-competent (circles) or not (squares); the dimer landscape is only composed of the structures with binding pocket. State transitions within each landscape are indicated by arrows; the individual associations and dissociations of the ligand (i.e., transitions between corresponding binding-competent states of the two landscapes), are not drawn here. To reduce the problem size (i.e., for coarse graining), the states are combined into macro-states defined by basins of attraction to local minima (filled squares and circles). Each secondary structure *x* is attracted to the local minimum that is the target of the ‘gradient walk’ starting from *x*, which corresponds to the energetically steepest descending walk. Such walks follow the solid arrows. The dashed arrows represent transitions to energetically better structures that are not maximally steep. Due to the construction of basins, there is generally no one-to-one correspondence between the macro-states of both landscapes. For example, while the monomer state *A* has a gradient transition, the corresponding dimer state $A^{\prime }$ has no gradient transition. Thus, $A^{\prime }$ constitutes a dimer macro-state without corresponding monomer macro-state. Similarly, there are macro-states of the monomer landscape without corresponding dimer macro-state: since the local minimum *E* is not binding competent, there is no corresponding dimer; while there is a gradient transition from *B* to *E*, $B^{\prime }$ is attracted to *D* (via gradient transition to $C^{\prime }$).

The kinetic approaches to single structure folding can be extended to RNA–RNA interactions and RNA–ligand binding, in principle. However, since interactions are inherently bimolecular, transition rates become explicitly concentration-dependent, which adds a layer of complexity. More precisely, bimolecular reactions, i.e., the binding steps, require explicit knowledge of concentrations. The situation simplifies again if the ligand is present in excess. In this situation, binding does not relevantly change the ligand concentration:

(6) $$K_{b}=\frac{\left[RNA:L\right]}{\left[RNA]\;[L\right]}\approx \frac{\left[RNA:L\right]}{\left[RNA\right]}\frac{1}{{\left[L\right]}_{0}}$$

and hence the energy landscapes for bound and unbound RNA conformations are simply shifted by a time-independent offset $\delta =-RT\ln{\left[L\right]}_{0}$ that depends on the ligand concentration only. It is possible therefore to generalize kinetic folding approaches discussed above with little changes [114,115]. In fact it suffices to model concentration-dependent association reactions of the type $R_{i}+L\to R_{i}:L$, where $R_{i}$ and $R_{i}:L$ denote the respective unbound and bound RNA in the same secondary structure *i*. This reaction is allowed whenever $R_{i}$ contains the necessary binding motif, and forbidden otherwise. Thus, it is plausible to assume that all allowed dimerization reactions occur at the same association rate. In this simplified scenario, the model can be composed of two RNA folding landscapes: an unconstrained one for the unbound state and one constrained to secondary structures containing the binding site for the bound state. As in the case of single molecule folding, the resulting model is amenable to analysis only after coarse graining; Figure 3 juxtaposes the coarse grainings of the two landscapes.

This analytical approach to RNA–ligand kinetics [114,115] enables computing of concentration- and time-dependent macrostate probabilities based on solving the master equation of the interaction process. Such methods bear the potential to scrutinize the kinetic properties of arbitrary riboswitch designs in silico. Exemplary, we discussed the interaction kinetics of the artificially designed theophylline riboswitch RS3 [23] at different concentrations and studied co-transcriptional effects; our findings suggest strong co-transcription influences on the function of RS3.

So far, the theory assumes a single binding motif. However, ligands are expected to bind to different sub-structures with different binding energies. In addition, there may be multiple binding sites. It is desirable, therefore, to extend the above approach to such more realistic scenarios. A second issue that is not yet solved in a satisfactory manner is the link between re-folding and co-transcriptional folding. The BarMap formalism [116] allows describing slow changes of coarse-grained landscapes by constructing maps between basins (i.e., macrostates) at consecutive time steps. It seems promising to combine this approach with the outlined landscape formalism for RNA–ligand interaction.

A key issue for the design of kinetically controlled switches is the availability of experimental data to dial in simulation time scales. Even where the mathematical formalism has been developed, in practice meaningful results crucially depend on additional experimental measurements of detailed kinetic prefactors and transcriptional speeds, which in turn depend on the specific biochemical environment of the binding process. In principle, such parameters could be measured in targeted experiments, but are currently unavailable for most model systems of interest.

### 3.3. Thermodynamic and Kinetic Design Principles

The computational models of RNA folding thermodynamics and kinetics, as outlined in the previous sections, make “in silico” design of RNA-based control systems possible. Naturally, computational design requires deep understanding of the working mechanisms in order to develop sufficiently accurate formal models; currently, modeling imperfection limits computational riboswitch design to reliably propose candidates for experimental validation.

From the modeling perspective, one distinguishes switches triggered by RNA-molecules and switches triggered by non-RNA ligands. For the former, it is reasonable to model the interaction, including binding motifs and energies, based on the RNA energy model. In contrast, the latter require empirical knowledge about the binding specificity of the ligand to the RNA, since in general this cannot be reliably computed. In the simplest case, one needs information on the sequence and structure of the binding pocket and the according binding energy. In these systems, ligand-specific aptamers are utilized as sensory platforms and are extended to construct the intended expression platform. In a typical scenario, the sequence of the aptamer is constrained and the binding-competent sub-structure is assumed to be known. Based on that, models of the switch’s OFF and ON state, e.g., regions that fold into terminator and anti-terminator structures in a transcriptional switch, are defined.

At the foundation of every design process, we require constraints and ranking criteria for riboswitch sequences. Simple hard sequence and structure constraints are, e.g., given by the ligand binding motif, which requires a specific sequence pattern and the possibility to fold into defined structure ‘patterns’. Additionally, there are thermodynamic constraints; for example, switching is observed only if the ligand-bound structure is sufficiently more stable than the ground state of the RNA, since otherwise the ligand can not significantly influence the ensemble of possible structures. Moreover, the binding-competent structure without the ligand must be less stable, such that adding the ligand changes the ensemble.

The ranking of designed sequences is particularly challenging, since generally there are multiple criteria for good designs, which cannot be weighted a priori. Moreover, many criteria, like tolerable deviations from the aptamer constraints or a terminator structure, are hard to formalize. For this reasons, such multi-objective optimization is commonly performed in a largely manual, non-automatized process by experts (see Section 4).

The thermodynamic model allows us, thereby enabling automatized rational design, to formalize important design criteria, like the ratios of RNA molecules in the ON and OFF state in the presence and absence of ligand in the thermodynamic equilibrium. For simplicity, assume for an activator system that ON and OFF state, as well as BINDING and NON-BINDING state of the aptamer are described (e.g., by structure constraints) such that the energies of the constrained subensembles in these states can be efficiently computed. We denote such energies as ΔG(BINDING), ΔG(NON−BINDING),ΔG(ON), ΔG(BINDINGandON), and so on, with the obvious meanings. Note that the RNA–ligand complex in ON-state, shortly denoted by “BOUND and ON”, has the energy $\Delta G\left(\mathrm{BINDING}\;\mathrm{and}\;\mathrm{ON}\right)+\theta_{L}$, i.e., it consists of the energy of the binding-competent RNA secondary structures in ON-state and the ligand–aptamer binding energy $\theta_{L}$.

In this model, good switching requires that the states BINDING and OFF are disjoint (or at least ΔG(BINDINGandOFF) is very high); the same holds for NON-BINDING and ON. Moreover, as discussed before, ligand-dependent switching behavior can be observed only if

(7) $$\begin{array}{cc} \Delta G(\mathrm{BOUND}\;\mathrm{and}\;\mathrm{ON})+x & \leq \Delta G(\mathrm{OFF}) \end{array}$$

(8) $$\begin{array}{cc} \Delta G(\mathrm{OFF})+y & \leq \Delta G(\mathrm{ON}) \end{array}$$

for sufficiently large energy differences *x* and *y*. These parameters directly control the concentration ratios of molecules in the different states, where values 0 require the ratio (between the states on the left and right side of the inequations) to be at least 1:1; higher values shift the ratios to the left side. More precisely, rewriting from the second inequation shows

$$\begin{array}{cc} \Delta G(\mathrm{OFF})-\Delta G(\mathrm{ON}) & \leq -y\\ \exp((\Delta G(\mathrm{OFF})-\Delta G(\mathrm{ON}))/RT) & \leq \exp(-y/RT)\\ \exp((-\Delta G(\mathrm{ON})/RT)-(-\Delta G(\mathrm{OFF})/RT)) & \leq \exp(-y/RT)\\ \frac{\exp(-\Delta G(\mathrm{ON})/RT)}{\exp(-\Delta G(\mathrm{OFF})/RT)} & \leq \exp(-y/RT)\\ \frac{\left[\mathrm{ON}\right]}{\left[\mathrm{OFF}\right]} & \leq \exp(-y/RT), \end{array}$$

where [ON] and [OFF] denote the equilibrium concentrations of ON and OFF state. Consequently, Inequation (7) constrains [BOUND and ON] to be at least exp(−x/RT) times higher than [OFF] and Inequation (8) constrains [ON] to be at least exp(−y/RT) higher than [OFF].

One can reasonably expect that such energy differences *x* and *y* are similar to observed energy differences in related functional natural (or verified artificial) riboswitches. Consequently, this tells us to minimize the deviation from such differences *x* and *y* in our designs, which directly translates to (multiple) objective functions for the switch design. To avoid more complicated multi-objective optimization, one commonly constructs a total objective function as a weighted sum of the single objectives. Finally, this allows to automatize the generation of riboswitch candidates, which is the core idea of tools like MODENA [117] and RNAblueprint [118]; the latter provides a flexible library to support design with arbitrary objective functions and search strategies.

In practice, objective functions are limited to efficiently computable features. Efficient folding algorithms enable applying a multitude of thermodynamic features, e.g., based on the energies—and thus probabilities—of almost arbitrarily constrained structure sub-ensembles in the thermodynamic equilibrium. However, this limits the integration of complex kinetic features like fast re-folding from one state to the other—corresponding to low energy transition paths, into the objective function. Nevertheless, simple kinetic features or fast approximations can be cost-effectively utilized. For instance, Flamm et al. [119] proposed estimating barrier energies as part of the objective function.

The high computational cost of kinetic analysis suggests to consider more complex kinetic criteria only in a generate and test approach for the filtering of optimized candidates. For example, —given sufficiently accurate parameters—the RNA–ligand kinetics model of the previous section can estimate ligand concentration-dependent refolding times; thus detecting kinetically trapped candidates.

While refolding speeds influence transcriptional and translational switches, kinetics is potentially decisive for co-transcriptionally controlled transcriptional riboswitches. During the riboswitch synthesis, certain sub-structures, like the aptamer or a terminator, will ‘pop up’ only after the transcription of complementary pairing sequences; in turn, initially stable structures in the 5${}^{\prime }$-end can be massively destabilized due to the later formation of conflicting downstream sub-structures. Modeling such effects, requires to fine-tune the speed of transcription with the remaining model parameters; e.g., if downstream structures destabilize the binding pocket, transcription must be sufficiently slow to allow binding. Consequently, assuming too slow transcription yields false positives; too fast, false negatives. As of yet, we are not aware of kinetic analysis methods for this specific combination of RNA–ligand interaction and transcription (e.g., extending [114,115] to co-transcriptional interaction as suggested in the previous section).

In transcriptional switches, where the mRNA is terminated in the inactive state, while read through in the active state, the switching state takes effect exactly once per molecule. In contrast, the molecules of translational switches can be switched forth and back between ON and OFF state—these switches are reversible. This introduces a further design objective exclusively for translational switches: sufficiently fast, reversible switching between the states. Thermodynamically, this means that neither state ligand-bound or ligand-free should be too stable. Kinetically, the refolding times directly impact the response times and must be sufficiently fast.

### 3.4. Fold Changes for Activators and Repressors

Impressive fold changes based on protein expression measurements have been achieved for recently published designs [120,121,122]. This is especially true for translation activating switches. Remarkably, the higher success with designing activators, is unsurprising by purely theoretical considerations.

Commonly, fold change *F* is defined as the ratio of the readouts in ON and OFF state, i.e.,

(9) $$F=\frac{<\mathrm{ON}>}{<\mathrm{OFF}>}.$$

Generally the fractions $p_{b}$ and $p_{u}$ of the respective bound and unbound switches depends on the efficiency and stability of the trigger molecule binding to the switch.

For activators, we assume high expression <H> if all molecules were bound, while the low expression <L> results if all molecules are unbound. Since, the OFF state is independent of the trigger, we expect reporter gene expression <L>. However, the ON state depends on the binding efficiency and stability, i.e., on the fractions of the bound and unbound molecules; thus, the gene expression in the ON state is a mixture of high and low expression. Consequently, the activation fold change $F_{\mathrm{act}}$ is calculated as

(10) $$F_{\mathrm{act}}=\frac{p_{u}\times <L>+p_{b}\times <H>}{<L>}.$$

For repression, the ON state is independent and the OFF state is dependent on the trigger binding efficiency. Moreover, unbound molecules cause expression <H>; bound molecules, <L>. Thus, the repression fold change $F_{\mathrm{rep}}$ is

(11) $$F_{\mathrm{rep}}=\frac{<H>}{\left(p_{u}\times <H>+p_{b}\times <L>\right)}.$$

**Figure 4:**
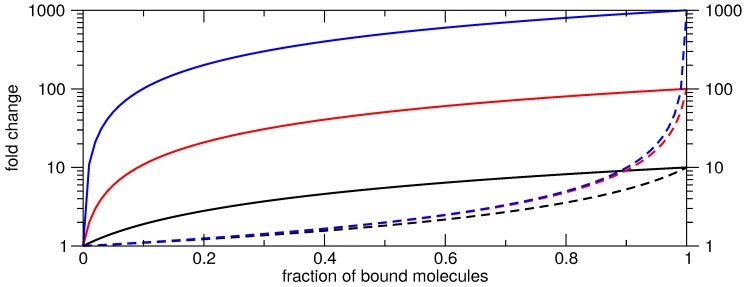
Achievable fold change for activating (straight) and repressing (dashed) switches depending on the fraction of bound molecules. The plots demonstrate that achieving the same fold change requires to bind a much higher fraction of molecules in represessing systems compared to activating systems. Different colors correspond to varying ratios of the assumed expression values in the OFF and the ON state (1:10, 1:100, 1:1000 are black, red and blue respectively). This ratio is also determined by the start at (0,1) and the end value at (1,y${}_{\max}$) of each curve. Note that the *y*-axis is shown in log scale.

Varying both ratios and simulating the resulting achievable fold change shows that it is rather easy to achieve high fold changes when activating even if only a small fraction of switches are bound, Figure 4. Here it is essential to have almost no expression in the OFF state and the higher the expression value in the ON state becomes the more pronounced the achievable fold change is already with a low fraction of bound molecules. For instance 10% of bound molecules gives already a two-fold activation even if the factor between low and high is only ten. As the OFF state is not concentration-dependent, i.e., only the switch needs to be trapped in this state, this design goal seems to be easy to achieve by computational design. The opposite gets evident for gene repression, Figure 4. Independent of the ratio between low and high gene expression values only a two-fold repression efficiency can be achieved although 50% of all molecules are bound. In order to get an about 10-fold repression efficiency approximately 90% of the molecules need to form the complex and a ratio of at least 1:100 of the low and the high expression value is needed. In summary the calculated fold changes need always be taken with caution and have to be analyzed with respect to the regulatory process, i.e., activation or repression.

## 4. Practical Designs

**Figure 5:**
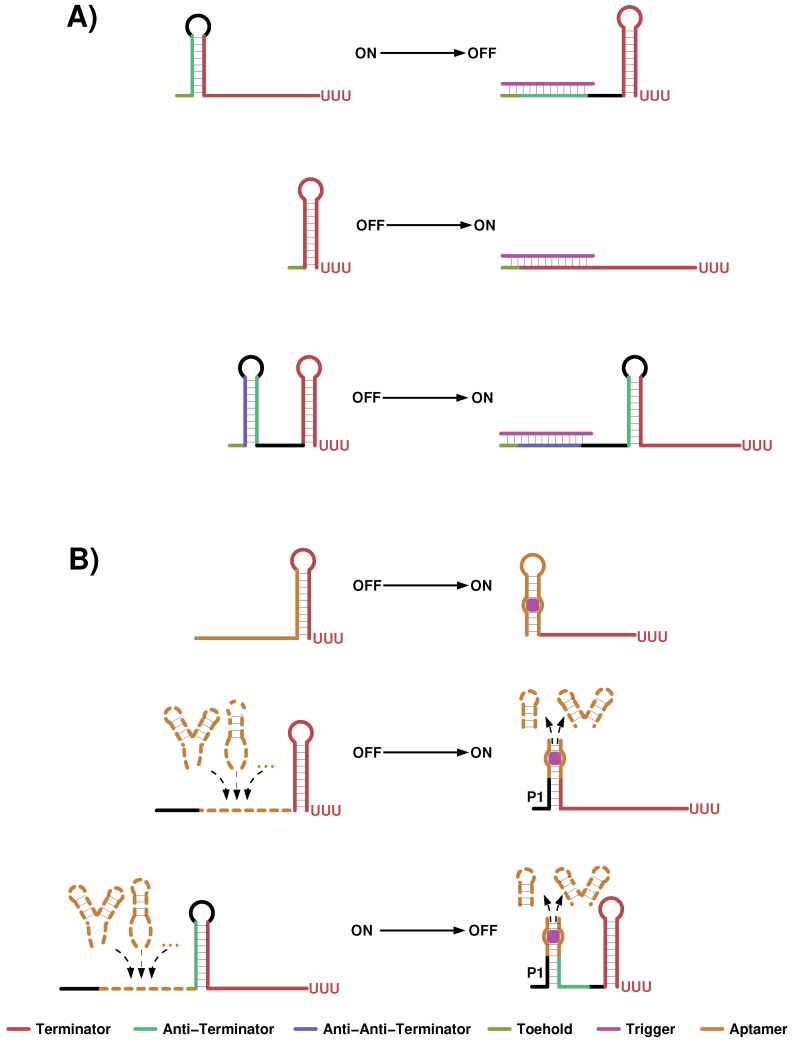
Cartoons to illustrate design ideas of recently published approaches to regulate transcription. (**A**) The sRNA based approach utilized by Chappell et al. [122] is shown. They started from a previously published transcription attenuator (top), where the unbound mRNA transcript folds into an anti-terminator structure. The latter is opened upon sRNA binding, which favors the formation of a transcription terminator. Two possibilities to convert this system into a transcription activator have been investigated by the authors. One is to directly target the 5${}^{\prime }$ part of the terminator stem (middle) and the second adds another layer of structural dependency by attaching an anti-anti-terminator sequence upstream of the initial construct. This causes the formation of an additional stem that sequesters the anti-terminator sequence and thereby ensures the proper terminator fold. (**B**) The aptamer based designs applied in [23,69,123] (top) and [124,125] (middle and bottom) are sketched. The first approach utilizes the stabilizing effect of ligand binding in order to avoid terminator formation, whereas the latter is based on the observation that many aptamers are closed by a typically short P1 stem, which is not essential for ligand recognition. Therefore diverse aptamer sequences can be attached to a generic P1 platform, which is further linked to a downstream terminator. When P1 is stabilized upon ligand binding terminator formation is either prohibited (middle) or facilitated (bottom).

**Figure 6:**
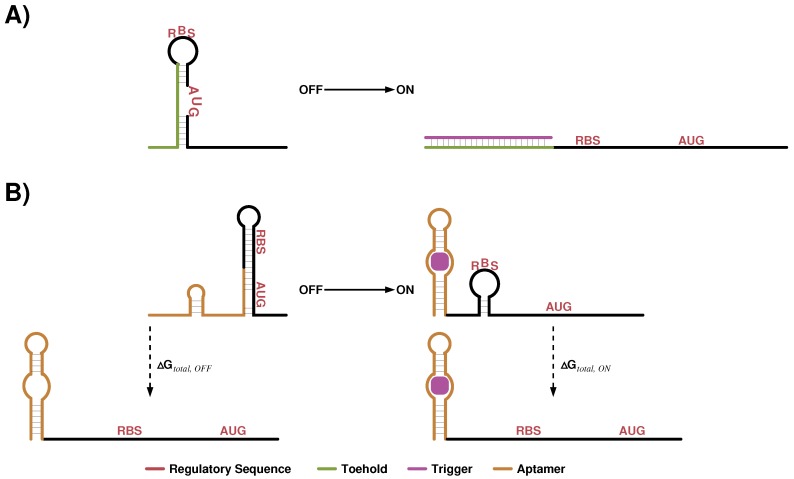
Cartoons to illustrate design ideas of recently published approaches to regulate translation. In (**A**) the approach utilized by Green et al. [120] is shown. The novelty of this design attempt was to place the RBS and AUG of the regulated downstream gene into loop regions of a structural element, which can be opened by binding to a toehold sequence. (**B**) depicts an aptamer based design developed by Espah-Borujeni et al. [121]. The Salis lab developed over recent years a detailed biophysical model to predict the energy $\Delta G_{total}$, which is necessary to open up structural elements that interfere with ribosome binding. This is applied in order to ensure that low gene expression is observed in the OFF and high values are detected in the ON state. Hence $\Delta G_{total,OFF}$ needs to be high and $\Delta G_{total,ON}$ low.

In the following we outline recent design attempts to regulate transcription and translation using sRNAs and non-RNA ligands as trigger, graphically summarized in Figure 5 and Figure 6.

### 4.1. Small Transcription Regulating RNAs

Chappell et al. [122] engineered sRNAs that interfere with the formation of an intrinsic terminator encoded upstream of a protein coding gene, see Figure 5A. They started from an already published system, where the host gene was fully transcribed if no sRNA was present because an anti-terminator sequence complementary to the terminators 5${}^{\prime }$ half interfered with its proper folding. Upon sRNA binding terminator structure formation was assisted. This behavior was inverted by extending the host gene to its 5${}^{\prime }$ end with a anti-anti-terminator sequence that enabled terminator formation without sRNA. Expressing an sRNA that is complementary to the anti-anti-terminator sequence now induced gene expression. Using this indirect mechanism Chappell et al. [122] observed an about 11-fold increase between measured protein levels of the OFF and the ON state. In order to remove one layer of structural repression the authors targeted in a more direct way the 5${}^{\prime }$ end of the terminator stem. For the three tested terminator sequences pbuE, T181 and AD1 fold activation of maximally 3, 19 and 94 has been observed, respectively. Quantitative PCR (qPCR) experiments were applied to complement the reporter gene fluorescence readout and to show that the regulation indeed happened on transcriptional level. Furthermore in vitro transcription-translation (TX-TL) reactions are utilized to confirm that the activating sRNAs directly regulate reporter gene transcription and not an off-target or nonspecific gene expression causes the observed effects. All tested constructs in this study are engineered combining known RNA sequences, e.g., attenuator and terminator sequences, and building their complements. In a followup publication the Lucks lab investigated the possibility to further increase the effect of the worst performing pbuE design [126]. Here again known parts such as promoter sequences of varying strength, stabilizing 5${}^{\prime }$ stems and sequence scaffolds taken from naturally occurring sRNAs were used in a plug and play manner. Applying this strategy the independently and repeatedly measured low initial fold-activation of 5.3 (±2.2) of the pubE construct [122] could be increased to 13.4 (±3.8). However for several constructs they observed unintended effects, e.g., base-pairing between the parts and the original pbuE sequences, that interfered with proper function. Hence this plug and play approach enabled the design of effective sRNA activated gene expression but still has it’s limitation in the predictability of the experimental outcome.

A few years earlier the group of Hervé Isambert designed *de novo* an antisense activation and an antisense repression system [71]. The design process was done by first drawing the global architecture of the system, i.e., drafting the state, in which the terminator is formed or the anti-terminator structure should occur, and the sequential order of the anti-terminator a spacer sequence and the terminator hairpin was determined. By making the terminator stable in absence or presence of a short oligo (15–16 nts in length) either an transcriptional ON or OFF switch was build. How the sequences are assigned to the individual parts is in a way uncertain from the publication itself. However the key feature of the design process is the simulation of co-transcriptional folding of individual candidates using the kinefold web service [127]. In an iterative approach the authors changed sub-sequences if unwanted structural intermediates occurred in the nascent RNA transcript until the simulation showed the appropriate behavior. To simulate the presence of the sRNA regulator its sequence was attached upstream of the 5${}^{\prime }$-UTR with a non-pairing linker. For the two presented and experimentally validated constructs the authors confirmed in vitro that they successfully designed a functional transcription repressor and an activator.

### 4.2. Transcription Regulating Riboswitch Design

In the design strategy for ligand sensing synthetic transcription regulating riboswitches, two approaches turned out to be successful, see Figure 5B. In the first, sensory and expression modules are overlapping and share a certain sequence part. As a consequence, the aptamer and the regulatory platform undergo structural changes, thereby adopting two alternative and mutually exclusive structures (ON and OFF state). Following this principle, several transcriptional riboswitches were constructed, based on aptamers for theophylline and tetracycline [23,69,123]. The design idea is rather simple: take a well studied aptamer, i.e., its structure and dissociation constant is known, and attach downstream sequences such that the 3${}^{\prime }$ half of the aptamer structure alternatively becomes part of a terminator stem. These downstream sequences are in essence a random spacer that should not interfere with proper aptamer folding while transcribed and a sequence that is perfect complementary to the aptamer’s 3${}^{\prime }$ half plus an eight nts long uracil track. When the ligand is present, the aptamer structure gets stabilized and terminator formation is inhibited. Without ligand terminator formation disrupts the aptamer structure and transcription is stopped. An essential first question was if functional terminators can be designed if their sequence is mainly determined by the used aptamer sequence. This question can be answered affirmatively at least for theophylline, tetracycline and streptomycin sensing RNA aptamers. For those aptamers, riboswitch constructs have been reported that showed repressed gene expression in the OFF state compared to a positive control (consisting of a plasmid expressing the reporter gene under the same promoter but lacking the aptamer and the terminator hairpin) [23,69,123]. Although transcription termination seemed to work, no functional riboswitch showing streptomycin-dependent induced gene expression could be generated [123]. The isolated transcription terminators of the theophylline-dependent riboswitches have been further investigated [69] and similar to Chen et al. [128] not only stem stability but also a fast loop closure showed to be indicative for proper function. However, an isolated functional terminator is not yet a functional riboswitch and the balance between a stable OFF state having less or ideally no read through and an ON state that is sufficiently stabilized upon ligand binding to inhibit terminator formation is needed. When analyzing the switching ability with respect to terminator stability this value alone seemed to be predictive at least in case of theophylline [23]. If the terminator is too stable, transcription is always stopped; in turn, if it is too weak, the terminator is never formed [23]. In the follow up study [69] in silico analysis indicated that kinetic traps might explain the exceptions to this rule. Such traps can be alternative local structures, which form during transcription thereby prohibiting fast terminator formation. In this case, even if the terminator structure is thermodynamically stable, it could not be formed in time, such that the switch appears always ON.

Using theophylline and tetracycline aptamers the best performing switches showed a fold change of 6.5 and 3.4, respectively. It appears that gene expression of the functional riboswitches in the ON state is close to maximal, when comparing it to the positive unregulated control, while the OFF state shows rather high leakage. This background level could be significantly reduced, when either multiple copies of the same riboswitch or different ones were placed in front of the reporter gene. The fold change of 6.5 of the best performing theophylline riboswitch could be further improved to 17 and 23, when utilizing two or three copies of it, respectively. For tetracycline the fold change improved from 3.4 to 7.5 and 9.5 for two and three copies. The improved performance directly results from having multiple points during transcription, at which the process can be terminated. However, a higher ligand concentration is necessary to bind multiple aptamer copies. In addition, heterogeneous tandem consisting of the best working theophylline and tetracycline riboswitches were constructed. When the theophylline switch was placed upstream of the tetracycline switch only the addition of both ligands induced gene expression by 10.4 fold as expected for a logical AND gate. However, when the order of the two riboswitches was swapped already theophylline alone induces gene expression. This shows that, even if the design of individual genetic devices was successful, combining them to higher order circuits is not trivial by far.

In addition to theophylline and tetracycline, Domin et al. [123] applied the approach also to design streptomycin sensing riboswitches but none of the four tested candidates showed a clear switching behavior. The manifold possible explanations for this failure range from a relatively low affinity of streptomycin and the RNA aptamer to structural features that are currently not taken into account by the design approach.

In many natural riboswitches, the aptamer fold is closed by a short helix P1 that separates the sensory and regulatory platform. Inspired by this non-overlapping organization, the Batey lab utilized the P1 helix to combine natural regulatory platforms with a variety of different (artificial as well as natural) aptamers, resulting in functional transcriptional OFF [125] and ON [124] riboswitches. Their first attempt was based on two primary criteria: (a) the two domains of a riboswitch must not overlap and (b) an easy way for in vitro and in vivo functional analysis must exist. The first is true for a subset of all known naturally occurring transcriptional OFF switches, which can therefore be easily split within the P1 helix into a sensory and a regulatory domain. For the second criterion, the authors argue that in vitro single-turnover transcriptional termination assays are applicable to rapidly screen riboswitch constructs and only implement those in vivo that show proper function beforehand. This, however, further reduced the set of suitable natural riboswitches as guanine-sensing riboswitches from *Bacillus subtilis* that previously had been reported to function in vivo did not work in the applied in vitro setup. To subsequently demonstrate that observed regulatory effects are a direct result of the designed riboswitches, Ceres et al. [125] used point mutations that are known to disrupt the sensing ability of the utilized aptamers. Three switching platforms, i.e., *metE* and *yitJ* naturally sensing SAM, and *lysC* that normally senses lysine, and seven natural as well as artificial aptamers that sense for instance guanine, 2-AP, FMN and theophylline were successfully isolated and assayed. In total 21 mix-and-match combinations have been investigated in detail, of which three represent the natural *metE*, *yitJ* and *lysC* riboswitches. Regulatory efficiency of all constructs was measured by titration. Corresponding in vitro experiments gave three essential parameters: $T_{50}$—the amount of ligand needed to observe half of the maximal possible response, $\%T_{max}$—the maximal observed termination efficiency and DR—the dynamic range of possible regulatory response as the difference between $\%T_{max}$ and its complement at rather low ligand concentration. Additionally, the equilibrium dissociation constant ($K_{D}$) was measured by isothermal titration calorimetry (ITC) of all isolated aptamers. The two combinations that resemble the natural occurring SAM riboswitches gave good correlation between $T_{50}$ and $K_{D}$ and had highest DR values as well as high $\%T_{max}$ in this combination. Especially from the first the authors concluded that both switches are under thermodynamic control and therefore the aptamer ligand binding process is fully equilibrated before the regulatory decision is made. Interestingly, for the natural lysine sensing switch combination the measured $T_{50}$ is about 3-fold higher than the $K_{D}$ and the DR as well as the $\%T_{max}$ are higher in chimeric combinations of sensing and regulating modules. This indicates that the aptamer might have insufficient time to bind the ligand before the decision of (anti-)terminator formation is made. For all 18 chimeric combinations, ligand-dependent regulatory effects were measured in vitro with varying efficiency and the selected aptamer domain predominates the achievable $T_{50}$ and $\%T_{max}$ values. Furthermore, two chimeric riboswitches were successfully tested in vivo. Finally, for a small set of selected riboswitch constructs Ceres et al. [125] tested, whether their switching ability can be altered by (de-)stabilizing the P1 helix as this structural element directly influences the switching behavior. As expected, starting from a functional switch extending the P1 helix turns it constitutively OFF, while shortening the stem results in a permanent ON. Strikingly, the performance of a switch that initially showed only poor switching ability due to a long and stable P1 helix could be improved by reducing sequence complementarity of individual P1 base pairs.

In order to apply the mix-and-match design strategy to design chimeric transcriptional ON switches, an explicit decoupling of the sensory and regulatory domains was necessary [124]. Similar to the implemented de novo design approach by Wachsmuth et al. [23], natural ON riboswitches show a significant overlap between the sequence essential to sense the ligand, i.e., the aptamer, and the sub-sequence that forms the regulatory element, i.e., the rho-independent terminator. Two show cases have been studied in detail. The first started with the *pubE* adenine switch of *B. subtilis*. Before decoupling the sensory and regulatory modules the authors improved regulatory efficiency of this riboswitch by deletion of 11 base pairs at the 5’ end of the reported sequence. Decoupling was then done by replacing a sub-sequence, not essential for ligand recognition, of the P1 helix. Again diverse aptamers were tested but only one was able to complement the isolated regulatory platform to a functional switch. All remaining chimeric constructs terminated transcription-independent of ligand concentration. Notably, shortening the terminator U-track by one or two bases made them responsive by increasing the dynamic range but not altering $T_{50}$ and $\%RT_{min}$ to much. The second show case started with *metH* SAH riboswitch from *Dechloromonas aromatica*. This particular riboswitch does by default not follow the structural constraints of the mix-and-match approach as the P1 helix is in the ligand bound state extended by the P4 helix, which forms a pseudoknot. The natural sequence was cut at two positions to remove the pseudoknot and to convert the eight nt long P4 helix into the conceptually essential P1 helix. The tested constructs showed a high read-trough, i.e., $\%RT_{min}$ above 20, which Ceres et al. [124] attributed to the relatively long and therefore presumably to stable P1 helix. Mutation of up to three bases and thereby shortening of the P1 helix to five base pairs resulted in chimeric switches with dynamic ranges similar to those of the other tested platforms. However, a rather high read through in absence of the corresponding ligands was observed. Improving terminator efficiency by replacing its loop sequence by UUCG tetraloop resulted in a chimeric riboswitch with an about 10-fold induction upon ligand addition. This loop replacement strategy in order to stabilize the terminator structure was also tested for one of the above described de novo designs Wachsmuth et al. [69]. There, the insertion of another commonly found GAAA tetraloop altered gene expression in both states and therefore the overall switching ability could not be improved. Moreover, when inserting the UUCG tetraloop the riboswitch lost its switching ability and ligand-independent high gene expression has been reported. While Ceres et al. [124] used natural building blocks to assemble chimeric riboswitches Wachsmuth et al. [23] de novo designed their constructs. Placing the same tetraloop into a naturally evolved or into an artificially constructed context seems to have a huge impact on the outcome. This is an observation that needs to be independently verified.

Both approaches, the de novo design by Mörl and colleagues and the mix-and-match strategy of the Batey lab, repeatedly indicated that aptamers or even the designed riboswitches regulate gene expression differently depending on the surrounding sequence and structure context. This makes it particularly difficult to forecast the switch’s performance, regardless of whether the starting point is a naturally occurring riboswitch evolved by nature or a artificial aptamer generated by SELEX.

### 4.3. Small Translation Activation RNAs

Similar to the work presented in Section 4.1, Green et al. [120] designed toehold RNA switches, where a toehold is an unpaired sub sequence, which is thereby accessible and a complementary sequence can easily bind to it. If complementarity of the two molecules is longer than just the toehold, structural elements of the RNA switch can be opened and others might be formed. This mechanism is widely used in DNA computing where typically multiple strands are added one after the other to model multiple states. Here we focus on two RNA strands only.

In [120] the switch is a cis-repressed mRNA where the RBS sequence and the AUG are located in loops of a longer sub-structure (Figure 6A). Putting these sequence constraints into loop regions reduces their impact on the design problem and gives complete flexibility to the optimization of the enclosing stems. A single stranded toehold sequence is placed upstream of the structural element. The trigger is an RNA sequence that is complementary to the toehold and the 5${}^{\prime }$ half of the switch’s stem loop structure. In summary, the OFF state is represented by the switch alone where RBS and AUG are sequestered and thereby translation is inhibited. Adding the trigger sequence causes opening of the inhibitory structure and enhances protein expression. Based on the sequence and structure constraints, optimized switch-trigger sequence pairs were generated with NuPACK. In silico, this tool optimizes the ensemble defect in order to increase the chance of well defined ON and OFF states in vitro as well as in vivo. Green et al. [120] initially generated a large pool of potential toehold switches, of which they selected a smaller subset by applying a Monte Carlo simulation to get orthogonally working devices, i.e., each trigger sequence should specifically regulate its designed switch partner only. This set showed a dynamic range of fold changes (*F*) getting up to more than 250 and nearly two thirds had values greater than 10. For a sub-set of 26 switch-trigger pairs they confirmed orthogonal functionality. One aspect of the publication was to investigate how the toehold switch performance varies if the reporter gene is changed. Three selected designs were tested with four different fluorescent reporters, i.e., cerulean, sfGFP, venus and mCherry. As expected, reporter signal strength varies depending on the reporter. Surprisingly, a toehold switch that performs best with venus (F≈80) showed worst performance when tested with mCherry (F≈15). Hence, caution is needed when experimental results are investigated and for instance correlations between calculated features and measured values are analyzed. The context, in which the designed construct is embedded, seems to be crucial and it might be a good design criteria to select for universally functional designs.

When designing toehold switches *de novo*, one would like to get best performing candidates only. For this reason, the authors investigated important features of the initial design idea. They varied loop sizes, closing base-pairs of the stem loop structure, toehold length and the length of complementarity between trigger and the 5${}^{\prime }$ half of the switch’s stem loop. For each of these features they found parameters that gave optimal fold change. In a second design run they adapted the initial sequence and structure constraints accordingly, designed and evaluated 13 toehold switches. For twelve of them a F≥270 was reported while the remaining one still achieved an impressive fold change of 33±4. To see whether the performance of a switch can be predicted based on thermodynamic features, the authors conducted R${}^{2}$ tests. The best R${}^{2}$ value was observed with $\Delta G_{RBS-linker}$, a thermodynamic quantity that evaluates the free energy of the switch’s sub-sequence starting at the RBS down to the first nt of the reporter gene coding sequence. This term estimates the energy needed by the ribosome to completely unfold the sub-sequence and to initiate translation when the trigger is bound. A more complex model of this process was described and applied for designing translational riboswitches, see Section 4.4. Green et al. [120] further showed that switch sequences can be generated to sense endogenous messenger RNAs and small RNA sequences in a cell, trigger sequences can be designed to synthetically regulate endogenous genes and multiple trigger-switch pairs can be combined to multiplex regulation or implement complex logical gates. This publication highlights many aspects of RNA design and exemplifies how potent a single design idea can be if it is successful when implemented in a cell.

### 4.4. Translation Regulating Riboswitch Design

Starting in 2009 Howard M. Salis and colleagues developed step by step a biophysical model to predict the translation initiation rate of a given mRNA sub-sequence, i.e., a 5${}^{\prime }$-UTR plus the first 35 nts (nt) of the open reading frame [121,129,130,131]. In total five energetic terms are combined into a total free energy

(12) $$\Delta G_{total}=\Delta G_{mRNA--rRNA}+\Delta G_{start}+\Delta G_{spacing}-\Delta G_{standby}-\Delta G_{mRNA},$$

where $\Delta G_{mRNA--rRNA}$ is the energy gain when the last nine nt of the 16S rRNA are bound to the mRNA sub-sequence, $\Delta G_{start}$ is the energy released when the tRNA anti-codon loop binds to the start codon, $\Delta G_{spacing}$ is a energy penalty that is added when the distance between the 16S rRNA binding site, which is the Shine Dalgarno sequence (SD), and the start codon is not optimal, $\Delta G_{standby}$ is the energy necessary to open structural elements upstream of the SD, and $\Delta G_{mRNA}$ is the energy that is necessary to unfold the mRNA sub-sequence. $\Delta G_{mRNA--rRNA},\Delta G_{start}$ and $\Delta G_{mRNA}$ are energetic terms that can be predicted with available tools such as the ViennaRNA package [82] or NuPACK [89]. The other two terms, i.e., $\Delta G_{spacing}$ and $\Delta G_{standby}$, have been determined using a learn-by-design procedure. The idea of this approach is to keep all parameters and the corresponding sequences of Equation (12) fixed and only change the sub-sequence and thereby the parameter of interest. To determine $\Delta G_{spacing}$ the authors varied the spacer length between 0 and 15 nt, measured reporter gene expression and fitted the data to a quadratic and a sigmoidal equation if the spacer is longer or shorter than the optimal 5 nt, respectively. Initially $\Delta G_{standby}$ was just the energy needed to unfold the four nt upstream of the SD. Using this estimate the predictive power of the biophysical model was rather low for long 5${}^{\prime }$-UTRs. Salis and colleagues hypothesized that $\Delta G_{standby}$ is not well captured by the model and investigated the effect of structural elements located upstream of the SD. They found that standby site modules, basically stem loop structures with a distal and proximal unpaired region can modulate translation initiation rates over 100-fold. $\Delta G_{standby}$ has been revised and the Salis group showed that ribosomal distortion, selective RNA unfolding and ribosomal sliding are essential to model translation initiation of mRNAs with long 5${}^{\prime }$-UTRs.

The high predictive accuracy of the biophysical model paved the way to design translation regulating riboswitches by making the translation initiation rate of the ON state high and of the OFF state low [121], see Figure 6B. Using statistical thermodynamics $\Delta G_{total}$ can be converted into the predicted translation initiation rate $r\propto \exp(-\beta \Delta G_{total})$ with the corresponding Boltzmann constant β=0.45±0.05 mol/kcal [129]. In a first step the authors showed that the models predicted activation ratio $AR_{max}=r_{ON}/r_{OFF}=\exp\left(-\beta \left[\Delta G_{total,ON}-\Delta G_{total,OFF}\right]\right)$ correlates well with in vitro measured luciferase expression of 15 previously published [132] theophylline-dependent riboswitches. Beside theophylline, aptamers that sense TMR, dopamine, thyroxine, DNT and fluoride were used to de novo design riboswitches and subsequently test them in vitro and/or in vivo. In brief, an aptamer sequence is placed between mutatable pre- and post-sequences. Downstream of the post-sequence that contains the RBS the reporter-coding sequence was attached. The optimization procedure applies a genetic algorithm that starts with an initial pool of random pre- and post-sequences. Subsequently, mutants are generated and evaluated until the design goal has been achieved. Evaluation of the translation efficiency of the individual sequences is done by calculating $AR_{max}$ or $\Delta \Delta G_{mRNA}=\Delta G_{total,ON}-\Delta G_{total,OFF}$ with the biophysical model. Using a diverse set of aptamers the authors realized that the predictive power of the biophysical model varies depending on the underlying sequence. They, therefore, adapted the model in order to account for ligand concentration dependence, stability of the mRNA:ligand complex and macromolecular crowding. The authors impressively demonstrated the versatility of their design method as the performance of 55% of the tested riboswitches were correctly modeled. As the authors state “(...) all models are imperfect (...)” of course the thermodynamic based biophysical model has its limitations. Currently the model does for instance not account for RNA degradation or kinetic parameters such as co-transcriptional folding. Beside these limitations of the theoretical model the authors pinpoint that the biological testing system itself restricts the achievable fold change when nanomolar ligand levels should be sensed. In principle the design approach by Espah-Borujeni et al. [121] can be easily adapted to design translation repressing riboswitches. However, a relatively large fraction of all riboswitch molecules in the solution need to be bound in order to achieve comparably high fold changes, see Section 3.4.

## 5. Summary

In this review we balanced out the theoretical background and practical aspects of riboswitch design. We not only focussed on the classical metabolite binding riboswitches but also broaden the definition to RNA binding and environment sensing structures. The summarized recent design attempts clearly indicate that RNA based switches are attractive biosensors which can be modeled in silico. Combining these individual RNA based sensors into higher order circuits is a challenging task. As described in [123] it is not straightforward to concatenate two individually functional riboswitches in to an presumably simple logical AND gate. This is just one reported example and we therefore think that context optimization in order to separate and thereby ensure functionality of individual RNA components is an essential next step in the research field of synthetic RNA biology.

## Abbreviations

The following abbreviations are used in this manuscript:

RBS	Ribosome Binding Site
mRNA	messenger RNA
sRNA	small RNA
UTR	untranslated region
nt	nucleotide
GFP	Green Fluorescent Protein
SELEX	Systematic Evolution of Ligands by EXponential enrichment
SD	Shine Dalgarno sequence
DFHBI	3,5-difluoro-4-hydroxybenzylidene imidazolinone
TO1	thiazole orange
HHR	hammerhead ribozyme
TX-TL	transcription-translation
ITC	isothermal titration calorimetry
